# Supplementary material for: Transgender fathering: Children’s psychological and family outcomes
Source: PLoS One. 2020 Nov 19;15(11):e0241214. doi: 10.1371/journal.pone.0241214 (PMC7676740; doi:10.1371/journal.pone.0241214)
Supplement: S1 Table — (PDF) [file pone.0241214.s001.pdf]

S1 Table. Kid-screen and attachment inventory of all participants who were eligible to perform the questionnaires

	Naturally Conceived Group (NC Group) N=28	*Conventional Donor Semen Insemination (CDSI Group) N=28*	*Transgender father and Donor Semen Insemination (TDSI Group) N=32*
**KIDSREEN (N_{NC}=13, N_{CDSI}=2, N_{TDSI}=10)**			
**Physical well being**			
mean (SD) [min;max]	60.4 (11;05) [47;73]	61.41 (16.67) [50;73]	54.34 (9.93) [43;73]
**Psychological well being**			
mean (SD) [min;max]	50.95 (6.67) [42;62]	54.2 (20.21) [40;68]	53.26 (5.77) [42;62]
**Moods and emotion**			
mean (SD) [min;max]	59.95 (11.56) [37;71]	57.41 (19.09) [44;71]	58.04 (8.93) [42;71]
**Self perception**			
mean (SD) [min;max]	50.96 (5.85) [41;70]	55.15 (20.69) [41;70]	54.29 (8.61) [41;60]
**Autonomy**			
mean (SD) [min;max]	49.26 (7.03) [34;56]	55.41 (18.87) [42;69]	49.57 (11.34) [37;69]
**Parent relation and homelife**			
mean (SD) [min;max]	50.95 (11.42) [36;66]	48.02 (25.23) [30;66]	49.79 (9.40) [41;66]
**Financial ressources**			
mean (SD) [min;max]	42.87 (9.96) [23;56]	59.6 (4.61) [56;63]	38.69 (12.28) [23;63]
**Social support and peers**			
mean (SD) [min;max]	47.61 (10.41) [34;71]	49.3 (1.33) [48;50]	42.66 (15.63) [9;71]
**School environment**			
mean (SD) [min;max]	52.98 (9.01) [41;74]	65.1 (12.3) [56;74]	54.08 (11.25) [45;74]
**Bullying**			
mean (SD) [min;max]	52.89 (7.98) [42;59]	58.85 (0) [59;59]	50.62 (7.48) [42;59]
**INVENTORY OF PARENTS AND PEERS ATTACHMENT (N_{NC}=11, N_{CDSI}=2, N_{TDSI}=8)**			
**Trust mother** ***(pr= 10-50)***			
mean (SD) [min;max]	45.18 (2.4) [41;49]	38.5 (16.26) [27;50]	43.12 (2.70) [38;47]
**Communication mother** ***(pr= 10-50)***			
mean (SD) [min;max]	34.63 (6.5) [21;45]	30.5 (9.19) [24;37]	33.75 (4.65) [28;40]
**Alienation mother** ***(pr= 8-40)***			
mean (SD) [min;max]	11.09 (3.14) [7;15]	13.5 (10.61) [6;21]	10.25 (3.77) [7;16]
**Trust father** ***(pr= 10-50)***			
mean (SD) [min;max]	43 (5) [31;49]	31 (26.87) [12;50]	42.75 (3.69) [38;48]
**Communication father** ***(pr= 10-50)***			
mean (SD) [min;max]	32.09 (5.58) [23;42]	26.5 (20.51) [12;41]	33.38 (4.10) [27;39]
**Alienation father** ***(pr= 8-40)***			
mean (SD) [min;max]	12.91 (4.76) [6;22]	16 (14.14) [6;26]	11.12 (4.05) [7;18]
**Trust peers** ***(pr= 10-50)***			
mean (SD) [min;max]	42.09 (3.99) [38;50]	47 (1.41) [46;48]	42.50 (6.61) [33;50]
**Communication peers** ***(pr= 8-40)***			
mean (SD) [min;max]	25 (6.99) [13;37]	19.5 (9.19) [13;26]	24.38 (7.95) [15;36]
**Alienation peers** ***(pr= 7-35)***			
mean (SD) [min;max]	14.73 (3.88) [7;20]	11 (5.66) [7;15]	14.38 (4.63) [9;23]

Pr= possible range
