# Supplementary material for: Transgender fathering: Children’s psychological and family outcomes
Source: PLoS One. 2020 Nov 19;15(11):e0241214. doi: 10.1371/journal.pone.0241214 (PMC7676740; doi:10.1371/journal.pone.0241214)
Supplement: S2 Table — (PDF) [file pone.0241214.s002.pdf]

S2 Table - Five Minute Speech Sample - NC Group vs TDSI Group				
	Naturally Conceived Group (NC Group) N=28	***Transgender father and Donor Semen Insemination (TDSI Group) N=32***	***Test***	***p (test)***
Mother **Expressed Emotion (N_{NC}=21, N_{TDSI}=30)**				
Low	**1** (5%)	3 (10%)		**p=0.034**
Limit	13 (62%)	15 (50%)	Chi2	
High	7 (33%)	12 (40%)		
**Mother Criticism (N_{NC}=21, N_{TDSI}=30)**				
**Low**	**17** (81%)	16 (53%)		**p=0.008**
**Limit**	3(14%)	9 (30%)	Chi2	
**High**	1 (5%)	5 (17%)		
**Mother Emotional Over Involvement (N_{NC}=21, N_{TDSI}=30)**				
Low	**1** (5%)	5 (17%)		**p=0.024**
Limit	14 (67%)	17 (57%)	Chi2	
High	6 (29%)	8 (27%)		
**Father Expressed Emotion (N_{NC}=19, N_{TDSI}=31)**				
Low	**7** (37%)	2 (6%)		**p<0.001**
Limit	10 (53%)	17 (55%)	Chi2	
High	2 (10%)	12 (39%)		
**Father criticism (N_{NC}=19, N_{TDSI}=31)**				
Low	**13** (68%)	15 (48%)		**p=0.002**
Limit	5 (26%)	13 (42%)	Chi2	
High	1 (5%)	3 (10%)		
**Father Emotional Over Involvement (N_{NC}=19, N_{TDSI}=31)**				
Low	**9** (47%)	5 (16%)		**p<0.001**
Limit	9 (47%)	17 (55%)	Chi2	
High	1 (5%)	9 (29%)		
