# Supplementary material for: Transgender fathering: Children’s psychological and family outcomes
Source: PLoS One. 2020 Nov 19;15(11):e0241214. doi: 10.1371/journal.pone.0241214 (PMC7676740; doi:10.1371/journal.pone.0241214)
Supplement: S3 Table — (PDF) [file pone.0241214.s003.pdf]

S3 Table - Five Minute Speech Sample - NC Group vs CDSI Group

	Naturally Conceived Group (NC Group) N=28	*Conventional Donor Semen Insemination (Cis-DSI Group) N=28*	*Test*	*p (test)*
**Mother Expressed Emotion (N$_{NC}$=21, N$_{CDSI}$=28)**				
Low	1 (5%)	9 (32%)		
Limit	13 (62%)	13 (46%)	Chi2	**p=0.021**
High	7 (33%)	6 (21%)		
**Mother Criticism (N$_{NC}$=21, N$_{CDSI}$=28)**				
Low	17 (81%)	23 (82%)		
Limit	3(14%)	4 (14%)	Chi2	p=0.230
High	1 (5%)	1 (4%)		
**Mother Emotional Over Involvement (N$_{NC}$=21, N$_{CDSI}$=28)**				
**Low**	1 (5%)	10 (36%)		
**Limit**	14 (67%)	13 (46%)	Chi2	**p=0.013**
**High**	6 (29%)	5 (18%)		
**Father Expressed Emotion (N$_{NC}$=19, N$_{CDSI}$=25)**				
Low	7 (37%)	8 (32%)		p=0.413
Limit	10 (53%)	15 (60%)	Chi2	
High	2 (10%)	2 (8%)		
**Father criticism (N$_{NC}$=19, N$_{CDSI}$=25)**				
Low	13 (68%)	20 (80%)		p=0.232
Limit	5 (26%)	5 (20%)	Chi2	
High	1 (5%)	0 (0%)		
**Father Emotional Over Involvement (N$_{NC}$=19, N$_{CDSI}$=25)**				
Low	9 (47%)	9 (36%)		p=0.359
Limit	9 (47%)	14 (56%)	Chi2	
High	1 (5%)	2 (8%)		
