# Supplementary material for: Transgender fathering: Children’s psychological and family outcomes
Source: PLoS One. 2020 Nov 19;15(11):e0241214. doi: 10.1371/journal.pone.0241214 (PMC7676740; doi:10.1371/journal.pone.0241214)
Supplement: S4 Table — (PDF) [file pone.0241214.s004.pdf]

S4 Table - Five Minute Speech Sample – Cis-DSI Group vs Trans-DSI Group

	Conventional Donor Semen Insemination (Cis-DSI Group) N=28	*Transgender father and Donor Semen Insemination (Trans-DSI Group) N=32*	*Test*	*p (test)*
**Mother Expressed Emotion** (**N_{CDSI}=28, N_{TDSI}=30**)				
**Low**	9 (32%)	3 (10%)		
**Limit**	13 (46%)	15 (50%)	Chi2	p=0.121
**High**	6 (21%)	12 (40%)		
**Mother Criticism** (**N_{CDSI}=28, N_{TDSI}=30**)				
**Low**	23 (82%)	16 (53%)		
**Limit**	4 (14%)	9 (30%)	Chi2	p=0.089
**High**	1 (4%)	5 (17%)		
**Mother Emotional Over Involvement** (**N_{CDSI}=28, N_{TDSI}=30**)				
**Low**	10 (36%)	5 (17%)		
**Limit**	13 (46%)	17 (57%)	Chi2	p=0.313
**High**	5 (18%)	8 (27%)		
**Father Expressed Emotion** (**N_{CDSI}=25, N_{TDSI}=31**)				
**Low**	8 (32%)	2 (6%)		
**Limit**	15 (60%)	17 (55%)	Chi2	**p=0.001**
**High**	2 (8%)	12 (39%)		
**Father criticism** (**N_{CDSI}=25, N_{TDSI}=31**)				
**Low**	20 (80%)	15 (48%)		
**Limit**	5 (20%)	13 (42%)	Chi2	**p=0.008**
**High**	0 (0%)	3 (10%)		
**Father Emotional Over Involvement** (**N_{CDSI}=25, N_{TDSI}=31**)				
**Low**	9 (36%)	5 (16%)		
**Limit**	14 (56%)	17 (55%)	Chi2	**p=0.016**
**High**	2 (8%)	9 (29%)		
