# Supplementary material for: Transgender fathering: Children’s psychological and family outcomes
Source: PLoS One. 2020 Nov 19;15(11):e0241214. doi: 10.1371/journal.pone.0241214 (PMC7676740; doi:10.1371/journal.pone.0241214)
Supplement: S5 Table — (PDF) [file pone.0241214.s005.pdf]

		Biologists				Endocrinologists				Med Students				Adult psychiatrists				Family Therapists			
**Rater number**		**1**	**2**	**3**	**4**	**1**	**2**	**3**	**4**	**1**	**2**	**3**	**4**	**1**	**2**	**3**	**4**	**1**	**2**	**3**	**4**
**Children who were conceived by donor sperm insemination in couples with a cisgender woman and a transgender man**	S1	1	-2	-1	-2	-2	2	2	-2	-1	-2	-2	-1	-2	1	-1	-1	1	-2	2	1
	S2	1	-2	2	2	-2	2	2	2	-1	1	1	-1	2	-1	-2	-2	-1	1	1	1
	S3	-1	-2	-2	-2	-2	-2	-2	-2	1	-1	-1	-1	-2	1	-2	-2	-1	-1	-1	-2
	S4	-1	-2	2	2	-1	-1	-1	-2	1	-1	1	-1	-1	-1	-2	-1	-1	-1	-1	-1
	S5	-1	-2	1	-2	1	1	-2	-2	-1	-1	-1	-1	-1	-1	-1	-1	1	-1	1	-1
	S6	-1	-2	-2	2	1	-2	-2	-2	2	1	1	-1	2	2	-2	2	1	1	-1	-1
	S7	1	-1	1	-2	1	-1	-2	-2	-1	-1	1	-1	-2	2	-2	-1	1	1	-1	-1
	S8	1	-2	-2	-2	-2	-2	2	2	2	2	2	-1	-2	1	2	-1	2	1	1	1
	S9	-1	-2	1	-2	1	-1	-2	-2	1	2	2	-1	-2	-1	-1	-1	-1	-2	2	-1
	S10	1	-2	1	-2	-2	-2	2	2	-2	-1	-1	-1	2	1	-2	-1	1	1	-1	1
	S11	-1	-2	1	-2	-2	-2	2	-2	1	-1	1	-1	-2	-2	-2	-2	-1	2	-2	-1
	S12	-1	-2	2	2	-2	-2	-2	-2	-1	2	-1	-1	2	2	-2	-2	1	-1	1	-2
	S13	1	-2	-1	-2	-2	2	2	2	1	-2	-2	-1	2	-2	2	2	2	1	2	2
	S14	-1	-2	-1	-2	-2	1	-2	-2	-1	-1	2	-1	-2	-1	-2	-1	1	-2	1	-1
	S15	-1	-2	-2	-2	-2	-2	-2	-2	-1	-1	-1	-1	-2	1	-1	-1	1	-2	1	-2
	S16	-1	-2	-2	-2	-2	-2	-2	-2	1	-1	-1	-1	-2	2	-2	-1	1	-2	-2	-1
	S17	-1	-1	1	2	-2	-1	-2	-2	-1	1	1	-1	2	1	-2	-2	-1	-2	1	-1
	S18	-1	-2	-2	-2	-2	2	-2	-2	1	-1	-1	-1	-2	2	2	-1	1	1	1	1
	S19	-1	-2	2	-1	1	-1	-1	-2	-1	-1	-1	-1	2	-1	-2	2	1	1	-1	-1
	S20	-1	-1	-2	-2	-2	-2	-2	-2	-1	-1	-1	-1	2	1	2	-2	1	-1	2	-1
	S21	-1	-2	-2	2	-2	-2	-1	-2	-1	-1	-1	-1	2	1	-2	-2	1	-2	-1	-1
	S22	-1	-2	1	-2	-2	-2	-2	2	1	-1	-1	-1	-2	2	-2	-2	-1	2	-2	-1
	S23	-1	-2	-2	-2	-2	-2	2	2	1	-1	2	-1	2	1	-2	-2	1	1	1	-2
**Naturally conceived control children**	C1	-1	1	1	2	-1	-2	2	2	1	-1	-1	1	-2	2	2	1	-1	-1	-1	-1
	C2	-1	2	2	-2	-1	2	2	2	1	-1	-1	1	-2	2	1	2	1	-2	-2	1
	C3	-1	2	2	-2	2	2	2	2	2	2	-2	1	2	-2	2	2	1	-1	1	2
	C4	1	2	2	2	2	2	-1	2	1	-2	-2	1	2	-2	2	2	1	2	-1	2
	C5	1	-1	2	-2	2	2	-2	2	1	-2	-2	1	2	-2	2	1	1	1	-2	1
	C6	-1	2	-1	2	2	2	1	2	2	2	-2	1	2	2	2	-2	-1	-2	-1	1
	C7	-1	2	-2	-2	-1	-2	-2	-2	-1	1	1	1	-2	2	-2	-2	-2	-1	1	-1
	C8	-1	-1	-1	-2	2	2	-2	2	1	1	1	1	-2	-2	2	-2	1	-1	-1	-1
	C9	-1	1	-2	-2	-1	-1	-2	2	1	1	1	1	1	1	-2	1	1	-2	-1	-1
	C10	1	1	-2	1	1	1	1	2	-1	1	-1	1	1	1	1	1	1	1	1	1
	C11	-1	1	-1	-2	-1	-1	2	2	-1	1	-1	1	-2	1	-2	1	-1	-2	-1	1
	C12	1	2	2	-2	2	2	2	2	-1	1	-2	1	-2	-1	2	1	-1	2	2	-1
	C13	-1	2	2	2	2	2	2	-2	1	-1	-1	1	2	-1	-2	2	-1	-1	2	1
	C14	-1	2	1	2	-1	2	2	2	1	-1	-1	1	2	-2	2	2	-1	-1	2	-1
	C15	1	2	2	-2	2	2	2	2	-1	1	-1	1	-2	1	2	1	-1	-2	2	1
	C16	1	2	2	2	2	2	1	2	-1	1	1	1	2	-2	2	2	1	2	2	1
	C17	1	2	-2	-2	-1	1	1	2	-1	1	-1	1	-2	1	1	1	-1	1	-1	1
	C18	1	1	1	2	2	-2	-2	2	-1	1	-1	1	2	-2	-2	1	-2	-1	1	-1
	C19	1	2	-1	2	2	2	2	2	-1	1	1	1	2	2	2	2	1	2	1	1
	C20	1	1	-2	-2	-1	2	1	2	-1	1	-1	1	-2	-2	1	-2	1	-1	-1	1
	C21	1	1	-1	-2	-1	1	1	2	1	-1	-1	1	1	1	1	1	1	1	-1	1
	C22	1	2	-1	2	2	-1	2	2	-1	1	1	1	2	1	1	1	1	1	1	1
	C23	-1	-1	2	2	2	2	2	2	1	1	-1	1	-2	2	2	-2	-1	-2	-1	-1
**Total per rater**		**-10**	**-13**	**-1**	**-24**	**-12**	**3**	**2**	**16**	**3**	**-1**	**-17**	**0**	**-1**	**12**	**-6**	**-8**	**10**	**-13**	**6**	**-5**
**Total per group of raters**		**-48**				**9**				**-15**				**-3**				**-2**			
**Permutation Test**		**p=0.04***				p=0.7				p=0.57				p=0.8				p=0.88			
